# Cubital Tunnel Release in a Patient With Parsonage-Turner Syndrome: A Case Report

**DOI:** 10.7759/cureus.84501

**Published:** 2025-05-20

**Authors:** Joshua L Dale, Frank Gerold

**Affiliations:** 1 Osteopathic Medicine, William Carey University College of Osteopathic Medicine, Hattiesburg, USA; 2 Orthopedic Surgery, Cornerstone Hospital, Edinburg, USA

**Keywords:** claw hand, comprehensive physical exam, cubital tunnel surgery, parsonage-turner syndrome, surgical nerve decompression

## Abstract

Parsonage-Turner syndrome (PTS) is a rare peripheral neuropathy characterized by the sudden onset of shoulder pain followed by muscle weakness and atrophy. This condition primarily affects the brachial plexus and may mimic or coexist with other compressive neuropathies. We present a case of a 39-year-old female with a prior diagnosis of PTS who developed progressive right-sided scapular and elbow pain, accompanied by numbness in the fourth and fifth digits of the right hand. Her symptoms had been ongoing for approximately three months. Physical examination revealed atrophy of the intrinsic muscles of the right hand, a resting claw deformity, and positive Wartenberg and Tinel’s signs at the elbow. Electromyography (EMG) was consistent with C8-T1 brachial plexopathy and ulnar nerve entrapment. Given the persistence of symptoms despite conservative treatment, surgical decompression via cubital tunnel release and anterior subcutaneous ulnar nerve transposition was performed. This case illustrates the rare overlap of PTS and ulnar nerve compression, suggestive of double crush syndrome. It underscores the importance of maintaining a high index of suspicion for superimposed compressive neuropathies in patients with PTS who present with focal neurological deficits.

## Introduction

Parsonage-Turner syndrome (PTS), also known as idiopathic brachial plexopathy or neuralgic amyotrophy, is a rare peripheral neuropathy characterized by inflammation of the brachial plexus [1,2]. It typically presents with the sudden onset of severe shoulder or upper arm pain, followed by muscle weakness, atrophy, and varying degrees of sensory disturbance. Symptoms are most commonly localized to the shoulder girdle and upper extremity, and may persist for several days to weeks [2-4]. The estimated incidence of PTS is approximately one in 1,000 patients presenting with complaints involving the neck, shoulder, or upper extremities, with a higher prevalence in males [5,6].

Management of PTS typically involves either conservative or non-conservative (surgical) approaches, depending on the severity and progression of symptoms [2]. In the case presented here, the patient demonstrated signs of ulnar nerve involvement at both the wrist and elbow, a pattern suggestive of double crush syndrome - a condition in which a single peripheral nerve is compressed at multiple sites along its course [7]. The patient experienced increased pain with Tinel’s sign at the elbow, which, combined with clinical evaluation, led to the decision to proceed with cubital tunnel release as a targeted intervention to release the entrapment.

## Case presentation

The patient is a 39-year-old female who initially presented to the clinic with right scapular pain as well as pain radiating down into the elbow, which had progressed to numbness in the third through fifth digits of the right hand. She stated that her symptoms began approximately three months prior while she was attempting to go to sleep. At the onset of symptoms, she was seen by her primary care physician and was started on oral steroids as well as muscle relaxants, which provided little relief. She denies any other systemic symptoms.

Physical examination was notable for intrinsic hand weakness along the fourth and fifth digits of the right hand, with intact sensation along the ulnar distribution of digits 4 and 5. There was diminished sensation along the median nerve distribution of the middle finger, as well as diminished sensation over the dorsal surface of the hand within the radial nerve distribution. Hand examination revealed atrophy of the first dorsal interosseus muscle with a resting claw deformity and positive Wartenberg sign. Further special testing exhibited Tinel’s test, which was positive at the elbow and negative at the wrist. Special tests are summarized in Table 1.

**Table 1:** Summary of special tests and other details.

Special test	Result	Location	Clinical implication
Wartenberg sign	Positive	Right hand	Suggests ulnar nerve dysfunction
Tinel’s sign	Positive	Elbow	Indicates ulnar nerve irritation at the elbow
Tinel’s sign	Negative	Wrist	No evidence of median nerve entrapment at wrist

Cervical spinal X-rays, including flexion and extension views, showed no abnormalities. An MRI of the cervical spine and right brachial plexus was ordered but was denied by the patient’s insurance. As a next step, an electromyography (EMG) was performed to evaluate the right upper extremity. EMG demonstrated reduced amplitude of the right median motor nerve (1.0 mV). The right radial motor nerve showed decreased conduction velocity (elbow - 4 cm, 35 m/s). The right ulnar motor nerve had prolonged distal onset latency (4.4 ms) with reduced amplitude (0.8 mV) and decreased conduction velocity (36 m/s). The right median sensory nerve showed reduced amplitude (8.8 µV) with decreased conduction velocity (mid-palm to 2nd digit, 39 m/s). The patient's results were conclusive for a right C8-T1 medial cord brachial plexopathy. Results of the EMG are summarized in Table 2.

**Table 2:** EMG findings summarized. EMG: electromyography

Nerve	Parameter	Result	Reference range	Interpretation
Right median motor	Amplitude	1.0 mV	>4.0 mV	Reduced
Right radial motor	Conduction velocity (elbow - 4 cm)	35 m/s	>50 m/s	Decreased
Right ulnar motor	Distal onset latency	4.4 ms	<3.5 ms	Prolonged
	Amplitude	0.8 mV	>6.0 mV	Reduced
	Conduction velocity	36 m/s	>50 m/s	Decreased
Right median sensory	Amplitude (mid-palm to 2nd digit)	8.8 µV	>15 µV	Reduced
	Conduction velocity	39 m/s	>50 m/s	Decreased

The patient was brought to the operating room and positioned supine. The operative arm was placed on a well-padded arm board in abduction with slight external rotation. Following administration of a regional block and general anesthesia, and after appropriate sterile preparation and draping, a longitudinal incision was made along the medial aspect of the elbow, following the course of the ulnar nerve.

Dissection was carried out through the subcutaneous tissue and investing fascia, with careful attention to preserve branches of the medial antebrachial cutaneous nerve. The fascia surrounding the ulnar nerve was identified and incised. The nerve was then released from all potential compression sites, including the arcade of Struthers, the ligament of Osborne, the cubital tunnel retinaculum, and the fascia of the flexor carpi ulnaris (FCU).

Upon intraoperative assessment, the ulnar nerve demonstrated limited mobility despite complete decompression. Given this finding, the decision was made to proceed with an anterior subcutaneous transposition of the nerve. The nerve was carefully mobilized and transposed anterior to the medial epicondyle. A soft tissue sling was fashioned using the patient’s subcutaneous tissue, and the nerve was loosely secured in its new position. Range-of-motion testing of the elbow through flexion and extension confirmed that the nerve remained free of tethering or undue tension in its transposed position. We then irrigated the wound with sterile saline and assessed proper hemostasis with the dropping of the tourniquet. The patient was then administered local anesthetic around the wound openings and irrigated thoroughly before closure.

The patient tolerated the procedure well without any surgical complications. They were transferred to the post-anesthesia care unit (PACU), where initial assessment revealed no new sensory or motor deficits. The operative arm was placed in a soft dressing with minimal compression, and the elbow was maintained in slight flexion to reduce tension on the transposed ulnar nerve. The patient reported adequate pain control, and there were no signs of wound dehiscence or other postoperative concerns. The patient was discharged the same day with detailed instructions regarding activity restrictions, arm elevation to manage swelling, and pain management protocols.

The patient is scheduled for a two-week follow-up for wound evaluation and suture removal. At that time, they will begin gentle range-of-motion exercises under the guidance of a physical therapist. A subsequent follow-up is planned at six weeks postoperatively to assess recovery, including evaluation of numbness or tingling in the ulnar distribution, grip strength, and any recurrence of symptoms.

At two-week follow-up, the patient reported almost complete alleviation of any ulnar, median, or radial nerve dysfunction in the right upper extremity. The patient demonstrated improvement of resting claw deformity as well. At six-week follow-up, the patient no longer complained of any nerve dysfunction in the right upper extremity, and the patient was now able to open and close the right hand with greater ease with almost complete resolution of claw deformity. Verbal consent was obtained from the patient to produce this report due to their unique presentation.

## Discussion

This case involved a cubital tunnel release in a patient previously diagnosed with Parsonage-Turner syndrome, who presented with symptoms more consistent with ulnar nerve entrapment at Guyon’s canal. Parsonage-Turner syndrome is a relatively rare condition, affecting approximately one in 1,000 individuals presenting with upper extremity or neck pain [5]. The syndrome is known for its variable clinical presentation.

Although the diagnosis of Parsonage-Turner syndrome had already been established, the patient’s primary complaint focused on ulnar-sided wrist and elbow pain, raising concern for a superimposed compressive neuropathy. Of particular interest in this case was the presence of symptoms in both the elbow and hand of the right upper extremity - findings more suggestive of double crush syndrome, in which a single peripheral nerve is compressed at multiple anatomical sites along its course [7]. In the case of our patient, the Parsonage-Turner syndrome may have created a predisposition for further mechanical compression at the cubital tunnel and Guyon's canal.

Our patient reported localized pain at both the cubital tunnel and Guyon’s canal, as well as signs of intrinsic hand muscle atrophy, particularly involving the lumbricals. A similar presentation was reported by Gonzalez-Alegre et al., where their patient exhibited ulnar neuropathy at the elbow in the context of Parsonage-Turner syndrome [4]. However, their case involved diffuse neurological deficits, including flaccid, areflexic paresis of all muscles in the left upper extremity, and mild hyperesthesia throughout the limb. In contrast, our patient demonstrated more focal findings, such as a positive Tinel’s sign at the elbow, pain localized to the wrist, and atrophy in the intrinsic hand muscles, with a resting claw deformity.

In a case described by Hussey et al., the patient underwent excision of benign inclusion cysts in the left, non-dominant thumb [3]. Five days postoperatively, the patient reported inability to flex the distal interphalangeal joint of the left index finger and the interphalangeal joint of the left thumb. Clinical examination revealed paralysis of the flexor pollicis longus (FPL) and flexor digitorum profundus (FDP) to the index finger, without associated pain or weakness in the elbow or shoulder [3]. This presentation differs significantly from the present case, where the patient demonstrated a more typical pattern of ulnar nerve entrapment localized at the cubital tunnel.

Differential diagnosis consideration

Several differential diagnoses were considered in this case. One important consideration was cervical radiculopathy, given the patient’s multiple upper extremity complaints, including hyperesthesia and some degree of muscle atrophy. However, the presence of a positive Tinel’s sign at the elbow and the predominance of ulnar distribution symptoms, such as atrophy localized to intrinsic hand muscles, suggested a more peripheral nerve entrapment.

Thoracic outlet syndrome (TOS) was also considered, as it can present with symptoms of ulnar nerve neuropathy and upper extremity pain [8]. However, this diagnosis was ultimately ruled out due to the patient's more focal symptoms and physical examination findings, which were not consistent with the diffuse and positional symptoms typically associated with TOS. Additionally, the presence of a resting claw deformity - an uncommon finding in patients with TOS - further supported an alternative diagnosis.

Rationale for surgical approach

Given the persistence of the patient’s symptoms despite an extended course of conservative management, along with focal findings on examination and imaging, surgical decompression of the ulnar nerve at the cubital tunnel was deemed appropriate. The decision to proceed with anterior subcutaneous transposition of the nerve was made intraoperatively due to continued restricted mobility of the nerve following standard decompression. This decision is not uncommon, as intraoperative assessment frequently guides the choice between in-situ decompression and transposition - approximately half of all ulnar nerve transpositions are determined during surgery based on intraoperative findings [9].

## Conclusions

This case highlights the importance of maintaining a high index of suspicion for compressive neuropathies in patients with concomitant Parsonage-Turner syndrome. When patients present to the clinic with focal findings, such as positive special tests, clinicians should consider additional or alternative diagnoses beyond Parsonage-Turner syndrome. Early recognition of these symptoms, followed by timely surgical intervention, can lead to significant symptom relief, even in the presence of an underlying brachial plexopathy.
